# Japanese Encephalitis Virus: The Emergence of Genotype IV in Australia and Its Potential Endemicity

**DOI:** 10.3390/v14112480

**Published:** 2022-11-09

**Authors:** John S. Mackenzie, David T. Williams, Andrew F. van den Hurk, David W. Smith, Bart J. Currie

**Affiliations:** 1 Faculty of Medical Sciences, Curtin University, Bentley, WA 6102, Australia; 2 CSIRO Australian Centre for Disease Preparedness, Geelong, VIC 3220, Australia; 3 Public Health Virology, Forensic and Scientific Services, Department of Health, Queensland Government, Brisbane, QLD 4108, Australia; 4 School of Medicine, University of Western Australia, Crawley, WA 6009, Australia; 5 Menzies School of Health Research, Charles Darwin University, Darwin, NT 0810, Australia

**Keywords:** Japanese encephalitis virus, flavivirus, JEV genotype IV, *Culex* sp. mosquitoes, ardeid birds, feral pigs, Murray Valley encephalitis virus

## Abstract

A fatal case of Japanese encephalitis (JE) occurred in northern Australia in early 2021. Sequence studies showed that the virus belonged to genotype IV (GIV), a genotype previously believed to be restricted to the Indonesian archipelago. This was the first locally acquired case of Japanese encephalitis virus (JEV) GIV to occur outside Indonesia, and the second confirmed fatal human case caused by a GIV virus. A closely related GIV JEV strain subsequently caused a widespread outbreak in eastern Australia in 2022 that was first detected by fetal death and abnormalities in commercial piggeries. Forty-two human cases also occurred with seven fatalities. This has been the first major outbreak of JEV in mainland Australia, and geographically the largest virgin soil outbreak recorded for JEV. This outbreak provides an opportunity to discuss and document the factors involved in the virus’ spread and its ecology in a novel ecological milieu in which other flaviviruses, including members of the JE serological complex, also occur. The probable vertebrate hosts and mosquito vectors are discussed with respect to virus spread and its possible endemicity in Australia, and the need to develop a One Health approach to develop improved surveillance methods to rapidly detect future outbreak activity across a large geographical area containing a sparse human population. Understanding the spread of JEV in a novel ecological environment is relevant to the possible threat that JEV may pose in the future to other receptive geographic areas, such as the west coast of the United States, southern Europe or Africa.

## 1. Introduction

**Figure 1:**
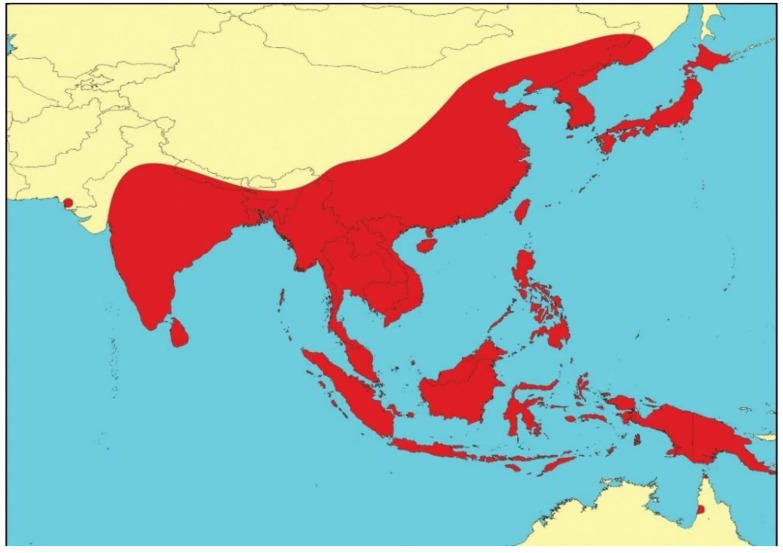
Geographical distribution of Japanese encephalitis virus prior to 2021. Map reproduced from van den Hurk et al. [6].

Japanese encephalitis virus (JEV), a mosquito-borne zoonotic flavivirus, continues to be the most important vaccine-preventable viral cause of human encephalitis in Asia [1,2]. It belongs to a subgroup of closely related viruses, including Murray Valley encephalitis virus (MVEV), St Louis encephalitis virus, and West Nile virus (WNV), which are often referred to as the Japanese encephalitis serological complex [3]. JEV circulates throughout much of Eastern and Southern Asia, from Maritime Siberia in the north, to South-East Asia and northern Australasia, India, Sri Lanka and possibly Pakistan in the south [4,5,6]—an area that encompasses about half of the world’s population (Figure 1). Epidemics of JE occur in temperate zones, with the majority of cases occurring in summer months; in tropical zones, JEV is endemic and transmission occurs year-round at a lower incidence. The virus is transmitted by *Culex* mosquitoes between Ardeid waterbirds (the maintenance hosts), such as the black-crowned night heron (*Nycticorax nycticorax*), and/or pigs (the amplifying hosts); humans and horses are incidental, dead-end hosts [6,7].

Epidemics of encephalitis had been recognized in Japan since 1871; the first large outbreak occurred in 1924, followed by another major outbreak in 1935. It was during the latter outbreak that JEV was first isolated in Japan from the brain of a fatal case [7]. This isolate, named the Nakayama strain, is recognized as the prototype strain of JEV. Thus, although the early history of JEV was in Japan, it is believed that JEV evolved from an ancestral virus in the Indonesia-Malaysia region [8,9], where it subsequently diverged into five genotypes (GI-GV), as distinguished by genome sequencing of the E or envelope gene [8,10]. Most of the early JEV isolates were later shown to belong to GIII, which together with the GI and GII viruses, were first described in 1990 [11]. Subsequently, GIV was described in 1992 [12], and GV was described in 2001 [8,13,14]. Of the five genotypes, GIV and GV are older and more divergent to the other three, with GV as the oldest and most divergent, followed in order by GIV, GIII, GII, and GI as the most recent [8,9,14]. GI viruses can be subdivided further on phylogenetic and epidemiological grounds into GIa and GIb [10,15]. The genotypes differ in their geographic distribution and in their disease; GIa, GII and GIV have been associated with tropical endemic disease, most often in children, whereas GIb and GIII are considered to be largely associated with seasonal, epidemic disease in temperate areas [10]. GIII was the dominant genotype in temperate areas from 1935 until the mid-1990s, but has over the past 2–3 decades been largely replaced by GIb [15,16,17,18,19]. Genotypes I, II and III are the most prevalent and account for 98% of the strains of JEV isolated from 1935 to 2009 [20], and thus most of our knowledge of the ecology, pathogenesis and control of JEV has been obtained from studies with JEV strains belonging to these three genotypes; relatively few investigations have been undertaken with GIV and GV viruses.

A number of differences have been observed between the genotypes, including antigenic heterogeneity and host diversity. Although JEV strains appear to comprise a single serogroup [21,22,23], various immunoassays clearly demonstrate a degree of antigenic heterogeneity occurring both between and within genotypes [24,25,26]. This is exemplified in cross-neutralization results between GIII and GI viruses [26,27,28,29]. Host diversity and replication efficiency in vector mosquitoes have also been suggested as reasons for genotype replacement from GIII to GIb [18,19].

### 1.1. Previous Emergence of JEV in North-Eastern Australia

**Figure 2:**
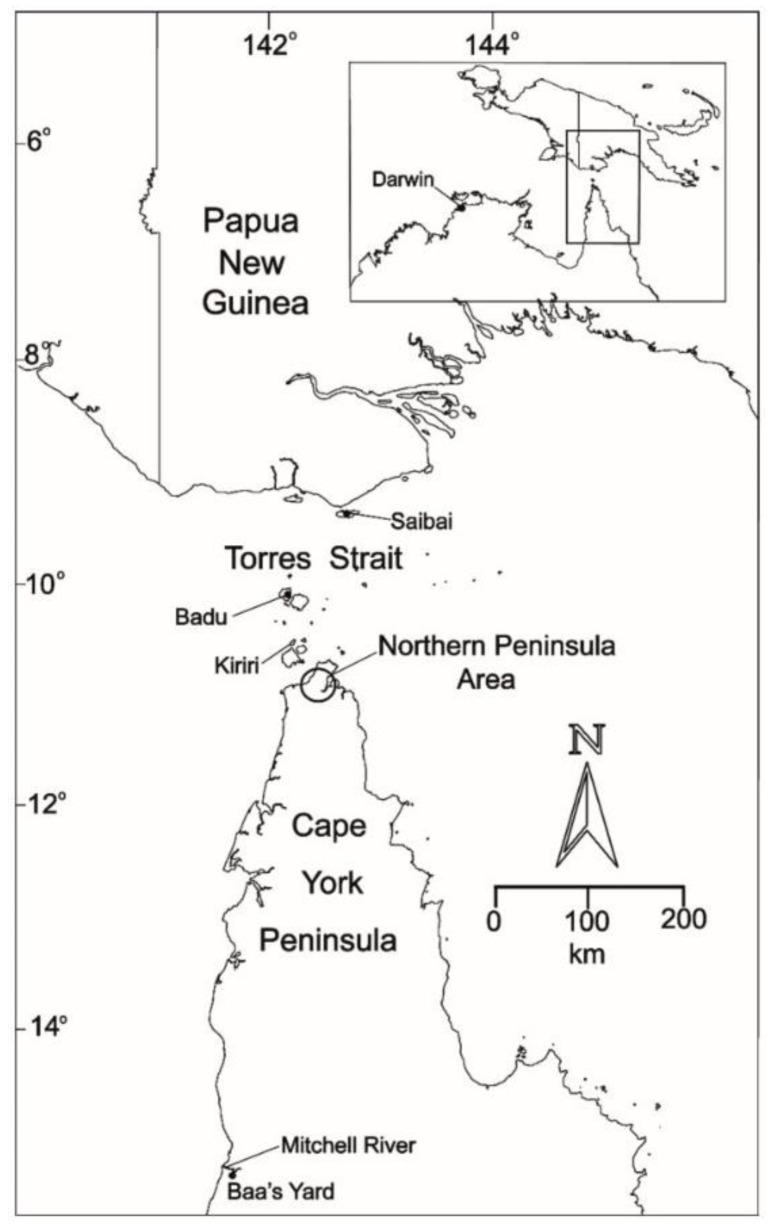
Map of Northern Australia and southern Papua New Guinea with the islands of the Torres Strait showing the areas referred to in the text of JEV incursions between 1995–2004. Reproduced from van den Hurk et al. [43].

JEV transmission in the Australian ecosystem had been first recognized from earlier incursions of JEV GII in the Torres Strait islands and the Northern Peninsula Area (NPA) of Cape York in north-eastern Australia in 1995 [30,31] and 1998 [32] (Figure 2), and JEV GI in 2000 [33] and 2004 [34]. These incursions were relatively widespread in the Torres Strait with infected pigs detected on a number of other islands in the central and northern Torres Strait [30,32]. Investigations clearly showed that the local vector mosquito species was *Culex annulirostris* and the amplifying hosts were domestic pigs kept in close proximity to human habitation. The origin of the incursions in 1995 and 1998 was found to be Papua New Guinea (PNG); genetically identical strains of JEV were isolated from *Cx. sitiens* subgroup mosquitoes trapped in Western Province of PNG [35]. The origin of the GI JEV in 2000 and 2004 remains unknown. There was no evidence to indicate that JEV of either genotype spread south beyond the mouth of the Mitchell River in the middle of Cape York (Figure 2). The occurrence of JEV in the Torres Strait and Cape York between 1995–2004 clearly demonstrated that JEV can be readily introduced into Northern Australia, and it highlighted the continued risk and vulnerability of the region to further JEV incursions of these or different genotypes. Over the following decade and a half, opportunistic serological sampling of pigs and horses in the Torres Strait by the Northern Australian Quarantine Strategy (NAQS) demonstrated that JEV was probably circulating seasonally in the Torres Strait, with 31 sero-positive animals of 1160 tested between 2008 and 2019, with positive animals reported in 2010, 2012, 2013, 2014, 2016, 2017 and 2019 (data compiled from NAQS reports in the Australian Health Surveillance Quarterly’s Quarterly Statistics). No human infections were reported, and no evidence of JEV circulation was detected from NPA or Cape York. It should be noted that these were single samples only, which provided evidence of past JEV exposure, and serological data cannot distinguish viral genotypes. The finding of occasional seropositive animals would suggest that JEV has either been endemic in the Torres Strait, or that the virus is reintroduced regularly from PNG. In 2020, the first indication of JEV from the NPA was reported with two sero-positive pigs and one sentinel cow; the latter was sero-positive in sera collected in April and May after being sero-negative in previous serum samples in January and February.

### 1.2. Aims of the Review

Until 2021, all Australian JEV activity had been restricted to the far north-east of Queensland, encompassing the Torres Strait and the NPA of Cape York. In early 2021, JEV was diagnosed in a patient in the Tiwi Islands of the Northern Territory and shown to be due to the rare genotype of JEV, GIV [36]; in early 2022, JEV GIV was identified as the cause of congenital malformations and fetal death in piggeries in south-eastern Australia, with a number of human cases also reported [37,38,39].

The purpose of this review is to describe the emergence and spread of JEV GIV in Australia—the first time this genotype has been demonstrated outside Indonesia—and to discuss the potential for it to establish in endemic transmission cycles in mainland Australia. As a virgin soil epidemic, it provides the opportunity to explore its origin and to document its spread and ecology in a novel ecological milieu in which other closely related flaviviruses of the Japanese encephalitis serological complex also occur [3,5]. In particular, it explores the probable vertebrate hosts and mosquito vectors involved in transmission cycles should JEV become endemic in Australia, and discusses effective surveillance and control strategies. The outbreak is believed to be the geographically largest virgin soil outbreak ever recorded for JEV and has relevance to the possible threat that JEV may pose to other potentially receptive areas such as the west coast of the United States [40,41], Africa and southern Europe [42], where competent mosquito vectors and potential vertebrate hosts also occur.

## 2. JEV Genotype IV and its Emergence in Australia

Prior to 2017, JEV GIV had the most limited distribution among the five genotypes of JEV, found only in Indonesia, with all seven recognized isolates having been obtained from wild-caught *Cx. tritaeniorhynchus*, *Cx. vishnui* or mixed pools of mosquitoes trapped between 1980 and 1981 in Java, Bali and Flores [12,44]. However, recent sequencing studies have indicated that a previously unrecognized 1979 isolate from Vietnam was also a GIV virus, but little is known about this isolate; it is believed to be from a human case, but no clinical information for the case or any travel history are available. It had been deposited into the World Reference Center for Emerging Viruses and Arboviruses by Dr J Landinshy from Hanoi (Personal communication from Dr K Plante, Assistant Director and Curator of the World Arbovirus Reference Center). There was a gap of 36 years from the 1981 isolates until 2017 when two GIV strains were isolated from pig sera collected in Bali [45], followed by a single isolate from a pool of *Cx. vishnui* mosquitoes collected in a pig shed in Bali in 2019 [46]. At about the same time, an Australian tourist to Bali was infected with a JEV GIV strain and later died in Brisbane [47]. This was the first confirmed human infection caused by a GIV virus. Thus, all other GIV viruses, except for the previously unrecognized isolate from Vietnam, have been from Indonesia. This changed in 2021 when a fatal case of JE occurred on the Tiwi Islands of northern Australia. A virus was sequenced and shown to also belong to GIV [36,48]. This was the first confirmed JE GIV case in which the infection had occurred outside Indonesia, and the first time an autochthonous GIV virus had been detected in Australia. Details of all known GIV viruses between 1979 and 2021 are shown in Table 1.

## 3. The Tiwi Islands Case of JE, and JEV in Northern and Eastern Australia

**Figure 3:**
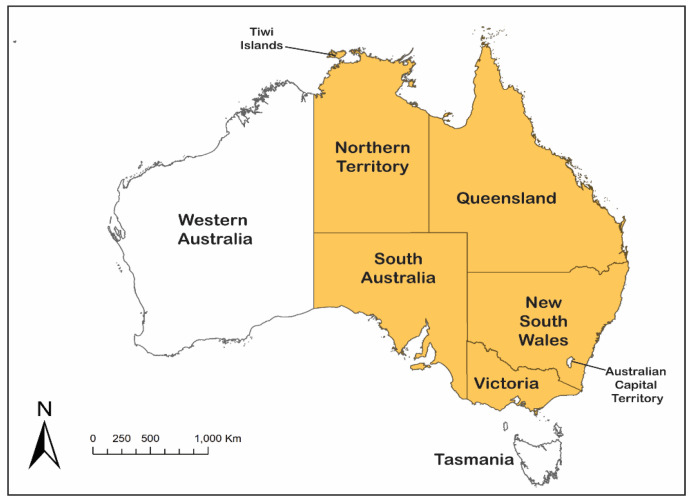
Map of Australia showing the Tiwi Islands and the States and Territories affected by JEV in 2021–2022 (orange shading). Human cases of Japanese encephalitis were reported from Northern Territory, Queensland, New South Wales, Victoria and South Australia; infected piggeries were reported from Queensland, New South Wales, Victoria and South Australia; infected feral pigs were reported from Queensland, South Australia and the Northern Territory; and isolations of JEV were obtained from mosquitoes trapped in Queensland, New South Wales and Victoria.

In February 2021 a 45-year-old female presented to her community health clinic on the Tiwi Islands, approximately 80 km north of Darwin in the Northern Territory (Figure 3), with a history of experiencing 2 days of fever and increasing confusion. Her comorbidities included end stage renal disease requiring dialysis and diabetes. She had not been outside the community in the preceding months [36]. She was evacuated by aeromedical retrieval to Royal Darwin Hospital. She had progressive neurological deterioration requiring admission to intensive care and intubation and ventilation, but she died during the third week of her illness. Analysis of cerebrospinal fluid (CSF) showed lymphocytic pleocytosis and elevated protein and magnetic resonance imaging (MRI) of the brain showed symmetrical hyperintensity in the thalami. The clinical, CSF and MRI findings were very consistent with those described for severe flavivirus encephalitis from MVEV, WNV and JEV [49], including the 2019 fatal imported GIV JE case from Queensland [49]. While MVEV is endemic in the Northern Territory, there had never been a case of MVE from the Tiwi islands. Given the potential analogy to the JEV incursions into the Torres Strait islands in north-eastern Australia [43], a diagnosis of either MVE or JE was considered most likely. Serology suggested flavivirus infection, but was unable to differentiate between MVEV or JEV. A complete JEV sequence was then obtained from post-mortem thalamic tissue, which was found to belong to GIV [36].

Given that this was the first documentation of locally acquired JEV in Australia outside of far north Queensland, surveillance was initiated to seek prior and ongoing evidence of JEV transmission in the Northern Territory. No evidence of JEV infection was found from retrospective molecular and serological testing of samples collected from feral pigs and buffalo on the Tiwi Islands in June 2021, which was undertaken by the NAQS program. However, several sentinel cattle sampled near Darwin between November 2020 and April 2021 were positive for JEV-specific antibodies, as well as a feral pig from Croker Island, approximately 100 km east of the Tiwi Islands (Dr S Fruean, NAQS, and Dr V. Bhardwaj, Berrimah Veterinary Laboratory, personal communication), but serology cannot distinguish genotypes.

Human infection with JEV is usually asymptomatic or causes a nonspecific mild illness, and encephalitis is estimated to occur in only 1:50 to 1:300 infected individuals [4]. Typically, JE mainly affects children in endemic areas, while adult cases are seen in travelers entering from endemic or epidemic areas, or where the virus enters a new area [50]. This is consistent with the two cases identified in Australia, which are the only two detailed descriptions of clinical cases of GIV JEV infection in humans. Both had clinical illness, radiological findings and a fatal outcome that is typical of severe encephalitic flavivirus infections [49,50].

**Figure 4:**
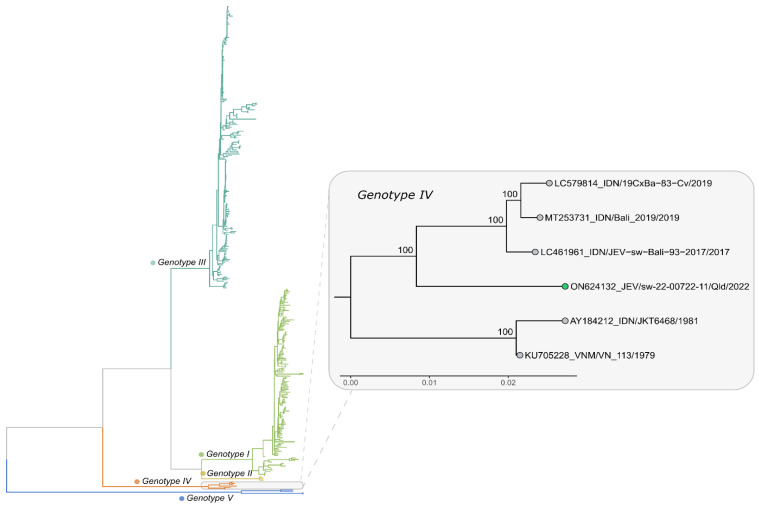
Phylogenetic tree showing the first JEV genome sequenced from an aborted fetus from a Queensland piggery in 2022 (green circle, inset) and its relationship to other available genotype IV whole genome sequences available from GenBank. The broader phylogenetic relationships of genotype IV with other JEV genotypes can be seen in the larger tree. The phylogenetic tree was estimated using the maximum likelihood method and a TN model with gamma rate heterogeneity, selected by IQ-TREE v.2.0.6. The results from 1000 bootstrap replicates are shown on the nodes and the scale represents the number of nucleotide substitutions per site.

Approximately 12 months after the Tiwi Islands case, in late February 2022, an outbreak of JEV was reported in eight pig farms in New South Wales (NSW), Victoria and Queensland, with fetal deaths, mummified fetuses and piglets born with shaking and fine nervous tremors [39]. As for the Tiwi Islands case, the virus detected from infected piggeries was also identified as belonging to a unique, yet related, lineage of GIV (Figure 4). It quickly became apparent that human cases of JE that had remained hitherto undiagnosed, including a fatality, were also occurring in temperate southern Australia [51,52], with the earliest onset of illness date being in late December 2021. On 1 March 2022, the JE outbreak in piggeries was reported to the World Organization for Animal Health (WOAH) by the Australian Chief Veterinary Officer [39], and on 4 March 2022, Australia’s Acting Chief Medical Officer formally declared the JE outbreak ‘A Communicable Disease Incident of National Significance’ [53]. The outbreak spread widely in pig farms in southern Queensland; central, western and southern NSW; northern Victoria; and eastern and South Australia (Figure 3), affecting over 80 pig farms [54]. A single rare case of JE in an alpaca was also reported in March from South Australia [54]. As of 1 November 2022, 42 confirmed and probable human cases of JE had been reported from the four States, including 7 fatalities [38]. This was consistent with the previously reported mortality of 15–20% [50]. However, there is insufficient data on population infection rates to determine whether the pathogenicity and neuro-invasiveness of the GIV strain differs from that of other genotypes. In a serological survey carried out in five towns in NSW, all of which had evidence of JEV-infected mosquitoes earlier in the year, there was evidence of JEV infection in approximately 1 in 11 of the 1048 participants, which indicates that JEV was prevalent in those areas and that a large number of people may have been infected [55]. It is important that further large, age-structured sero-epidemiological studies are undertaken over a broader area to determine the incidence and geographic spread of asymptomatic infections.

Surveillance activities in northern Australia were escalated by NAQS in April 2021 following the Tiwi Island case with increased animal testing, especially of feral pigs, and expanded further with the 2022 outbreak in the eastern Australian states. This expanded surveillance has led to the recent finding of JEV in over 50 feral pigs in the northern regions of the Northern Territory including the Tiwi Islands [56], as well as in feral pigs on the Cape York peninsula. Evidence of prior exposure to JEV was also found in feral pigs sampled in the North East pastoral region of South Australia [57].

When and where the virus was introduced and how and where from will likely never be known, although the wide geographic spread of the virus strongly suggests that the virus has been circulating for a number of months in Australia, if not years, prior to the 2022 outbreak. Similarly, whether it was the initial clinical case in the Tiwi Islands that gave rise to the outbreak in eastern Australia, or whether the Tiwi Islands case represented spread from prior initial JEV incursion elsewhere in northern Australia or a second incursion from the same overseas source may also remain unknown. Phylogenetic analysis showed that the 2021 Tiwi Islands JEV was closely related (>99.7%) to the 2022 south-east Australian outbreak strain [48]. The detection of JEV in mainland Australia, particularly in temperate areas of south-eastern Australia, caused very considerable alarm in the medical, agricultural and environmental sectors, and concern at the lack of any warning from surveillance activities. There is also concern at the potential risk of the virus becoming endemic in northern, tropical Australia, although detection in feral pig populations provides evidence that this may have already occurred. To understand the potential risk of endemicity, it is important to know the probable vertebrate hosts; the vector status of Australian mosquito species; the pathogenic potential of the virus; and the possible control measures available to limit human, porcine and equine infections.

## 4. Probable Vertebrate Hosts of JEV in Australia

The major vertebrate hosts of JEV are ardeid birds and pigs [58,59]. Ardeid birds, especially the black-crowned night heron (*Nycticorax nycticorax*) and various species of egrets, are believed to be the maintenance or reservoir hosts in Asia [59,60,61,62]. Related ardeid species also occur in Australia, such as the Nankeen night heron (*Nycticorax caledonicus*), plumed egret (*Ardea intermedia plumifera*), little egret (*Egretta garzetta*) and white-faced heron (*Egretta novaehollandiae*), and play a similar role for the closely related flaviruses, MVEV and WNV [63,64,65,66,67]. Serological results suggest that they are most likely to be major maintenance hosts for JEV. A number of other species of water birds have also been implicated as potential hosts of MVEV and WNV on serological grounds [66], including magpie geese (*Anseranas semipalmata*) [67], a waterbird that occurs in large numbers in northern Australia and southern PNG, ducks such as the wandering whistling-duck (*Dendrocygna arcuata*), and various species of cormorants and ibises, which are also possible hosts for JEV. While many studies have reported serological evidence of JEV exposure in ducks, their role remains uncertain, but ducklings, both domestic and wild, may play a minor role in outbreaks as experiments have demonstrated transmission between ducks and from ducks to chickens by *Cx. tritaeniorhynchus* [68], but peak viraemia decreases with age between 2 and 42 days [69].

**Figure 5:**
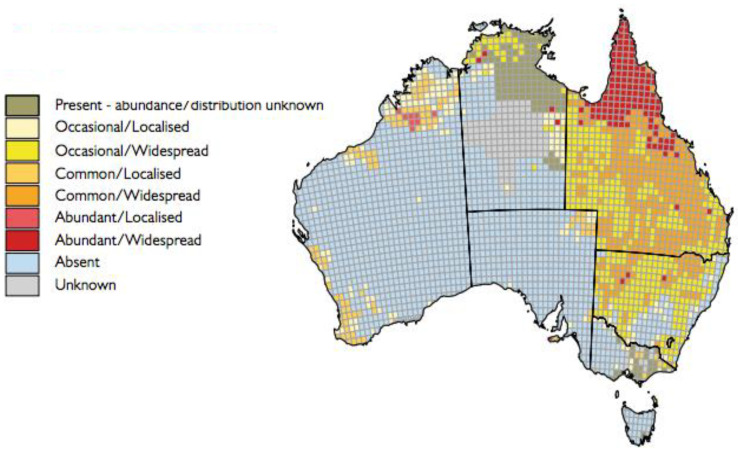
Map of Australia showing the occurrence, distribution and abundance of feral pigs (*Sus scrofa*) in Australia. Map generously provided by Invasive Animals Ltd. (Canberra Bruce, Australia)/Centre for Invasive Species Solutions.

Pigs are the major amplifying hosts in epidemic and endemic regions; they have a high natural infection rate and develop high levels of viraemia [4,7,20,70,71,72]. The close proximity of domestic pig breeding to human residential areas increases the risk of human exposure to JEV, resulting in an increased spillover to humans [19,73]. In Australia, both wild and domestic pigs would be expected to play a significant role in JEV ecology. Pigs were the amplifying host in the outbreaks of JEV in the Torres Strait and North Queensland in 1995–2000 [30,33]. Feral pigs are a major pest species in many parts of Australia, with approximately 4 million animals estimated to occur across about 45% of Australia’s land mass [74], significantly less than the 13.5 million animals previously reported [75], with the highest abundance in north-eastern Queensland, especially Cape York [74] (Figure 5). Increasing serological and virus isolation data indicates that they would be major vertebrate hosts for JEV in Australia. The domestic pig industry is centered in eastern, south eastern and south-western Australia, with most commercial piggeries located in New South Wales. There are approximately 4300 registered pig production sites in Australia with a population of about 2.4 million pigs at any one time [76], but relatively few commercial piggeries are located in the tropical north of Australia. In addition, an unknown number of unregistered pigs are kept in ‘backyard’ conditions in urban and peri-urban areas.

Other than humans, the only mammals that are known to develop clinical disease after infection with JEV are pigs and equids [4,58], with occasional signs of disease in cattle [58,77], but most infections are generally subclinical or mild. The South Australian alpaca JE case represents the first known report of JE in this species, for which there have also been reports of unusual cases of neurological disease following infection with WNV, which is closely related to JEV, in North America [78,79]. Humans, horses and alpacas are regarded as dead-end hosts [7,54]. Many domestic and wild animal species have been shown to have asymptomatic infections and do not appear to participate in natural transmission cycles [58,77,80]. An exception may be bats; both Megachiropteran and Microchiropteran bats have been implicated as possible reservoir hosts in JEV ecology on both serological grounds and from virus isolation [81,82], and may participate in the natural ecology of JEV [83,84,85,86,87,88].

Megachiropteran fruit bat species appear to be good candidates for participating in transmission cycles. Studies in India had demonstrated that *Cx. bitaeniorhynchus* and *Cx. tritaeniorhynchus* mosquitoes that were allowed to feed on JEV-infected greater short-nosed fruit bats (*Cynopterus sphinx*) became infected, and were then able to infect chickens. These studies demonstrated JEV transmission between bats, from bats to chickens, and from chickens to bats [88]. More recently, strong evidence suggests that lesser short nosed fruit bats (*C. brachyotis*) may act as reservoirs of JEV in the absence of pig breeding [86] in West Kalimantan, Indonesia, and this could also be the case for *Pteropus* sp. [87]. There are four species of pteropid bats or flying foxes in eastern and northern Australia: the little red flying-fox (*Pteropus scapulatus)*, the spectacled flying-fox (*P. conspicillatus)*, the black flying-fox (*P. alecto)* and the grey-headed flying-fox (*P. poliocephalus)* [89]. Little red flying-foxes have the widest geographic distribution in Australia, ranging from Victoria in the south to tropical northern Queensland and west to Shark Bay in Western Australia [90]. They, and other flying fox species, are becoming increasingly urbanized with most roosts in human modified landscapes [91,92]. They occur in large camps of tens of thousands to hundreds of thousands of animals [93], and are highly mobile [94]. Experimental infection of black flying foxes showed that they could be infected with JEV after being bitten by infected *Cx. annulirostris* mosquitoes, and were subsequently able to infect recipient mosquitoes in the absence of detectable viraemia, which demonstrates that they could also potentially participate in JEV transmission cycles [95].

The relevance of marsupials in JEV ecology is uncertain. Studies carried out at the Australian Animal Health Laboratory had shown that juvenile eastern grey kangaroos (*Macropus giganteus*), Tammar wallabies (*Notamacropus eugenii*) and agile wallabies (*Notamacropus agilis*) did not support JEV replication, although the latter displayed a brief low level viraemia in some animals. Brush-tailed possums (*Trichosurus vulpecula*) did develop a viraemia, and infectious JEV could be recovered over several days, which suggests that brush-tailed possums could potentially act as maintenance hosts [96].

## 5. Probable Mosquito Vectors in Australia

The major vectors of JEV in Southeast Asia and the Indian sub-continent are *Cx. tritaeniorhynchus* mosquitoes, with other important vector species in specific areas, including *Cx*. *gelidus*, *Cx. bitaeniorhynchus*, *Cx. vishnui* and *Cx. pseudovishnui* [6,97]. These species are either absent or have a limited distribution in Australia, thus other species would be expected to drive JEV transmission. Based on repeated isolates in the field, high transmission rates after experimental infection and host feeding patterns, *Cx. Annulirostris* is considered the primary vector of JEV in Australia [31,98,99,100]. This species is widely distributed in Australia and high population densities associated with La Niña rainfall events have historically been associated with outbreaks of MVEV and WNV in SE Australia [101,102,103].

Other *Culex* spp. Could also be involved in JEV transmission cycles in Australia. Both *Cx. Sitiens* and *Cx. Palpalis* are related to *Cx. Annulirostris*, have potentially yielded isolates in the field, and are able to transmit the virus at high rates in the laboratory [35,104,105] (L. Melville, unpublished data cited in [105]). Based on previous JEV studies, vector competence with local, indigenous populations and association with MVEV and WNV, Australian members of the *Cx. pipiens* group such as *Cx. quinquefasciatus*, *Cx. molestus* and *Cx. australicus*, as well as *Cx. bitaeniorhynchus*, may also be involved in transmission [106,107,108].

Two major vectors of JEV overseas have become established in Australia in the last 25 years. *Cx. gelidus* was first recognized in Brisbane and Mackay in 1999 [109] and has yielded isolates of JEV and shown to be a highly efficient laboratory vector [110,111]. A closely related JEV GIV strain to the Tiwi Islands virus (98% nucleotide identity) was also detected in *Cx. gelidus* trapped in Morobe Province, PNG, in 2019 (D.T. Williams, unpublished results). This species has subsequently been shown to have a widespread distribution in northern Australia [112] and relatively large larval populations have been associated with wastewater and effluent ponds [113]. Twenty years after *Cx. gelidus* was detected, established populations of *Cx. tritaeniorhynchus* were found in the Northern Territory in early 2020 [114]. Whilst population densities of this species are currently low (N. Kurucz, unpublished) it has the potential to spread, and with *Cx. gelidus*, it will no doubt add to the suite of potential vectors of JEV in Australia.

The role of other mosquito genera in transmission cycles of JEV is less defined. For instance, *Aedes vigilax*, *Ae. notoscriptus* and *Ae. albopictus* were able to transmit the virus in the laboratory, but transmission rates were considerably lower than *Culex* spp. [104,115]. High population densities of these species could offset their lower vector competence. Other *Aedes* spp. that occur in relatively high densities, such as *Ae. sagax*, *Ae. normanensis* and *Ae. vittiger* could have been involved in the recent emergence of JEV, particularly at the start of the transmission season before populations of *Cx. annulirostris* increased. Indeed, vertical transmission via the desiccation-resistant eggs of *Aedes* spp. [116,117], in conjunction with overwintering parous female *Culex* spp., could provide a mechanism for virus overwintering.

## 6. Geographic Spread of JEV in Australia

The origin of the JEV GIV strains that caused the case of JE in the Tiwi Islands and the outbreak in eastern Australia remain unknown, but their origin was probably an enzootic focus in the Indonesian archipelago, Timor Leste or possibly in PNG. At this time, no focus of activity has been found outside Indonesia, with most recent activity centered in Bali, except for the single isolate of JEV GIV from Morobe Province, PNG. Like some of the other major flaviviruses, JEV has a propensity to spread and establish in new areas [5,6,118]. When the area has not had any previous JEV activity, outbreaks affect all ages, but the most severe disease is most frequently in those over 60 years of age [119]. Virus spread has been associated with a number of factors including population growth, deforestation followed by the development of new irrigated crop growing areas that provide suitable habitats for mosquito larvae and for attracting ardeid birds, and the increase in intensive pig rearing [20,120,121] that has occurred over recent years in parts of Asia. JEV may be introduced into new areas by infected wind-blown mosquitoes [122,123,124,125,126]; through infected vertebrates, particularly nomadic or migratory birds, such as herons, egrets and magpie geese [66,127,128]; and possibly through infected pteropid bats [129]. The potential movement of nomadic or migratory birds between PNG and Australia provides a ready conduit for virus dispersal to Australia [130,131,132,133]. Every year occasional Asian vagrants are also picked up in northern Australia as evidence of over-shoot migration, which is potentially wind-assisted (S.T. Garnett, personal communication), but their relevance to virus dispersal is difficult to quantify. Experimental peak viraemia in ardeid birds is usually between 2–6 days post infection [61,62,63,134], and is highest in younger birds, so the period between infection and arrival in Australia needs to be considered as part of any risk analysis. Long distance mosquito movement can occur by means of abiotic mechanisms, such as commercial air or sea transport, as may have occurred in the spread of JEV to Guam [135], to Saipan [136] and more recently to Angola [137] and possibly to Italy [138,139,140]. Air or sea transport may also have been the mechanisms by which *Cx. tritaeniorhynchus* mosquitoes were introduced into northern Australia, although wind-blown mosquitoes have also been implicated [114]. “Airport dengue” has been described from Darwin, Northern Territory, attributed to virus infected mosquitoes harboring aboard an international flight, possibly from Bali [141]. Long distance, wind-blown mosquitoes during the monsoonal season have also been suggested as the means by which JEV moved from PNG to the mouth of the Mitchell River in Cape York in 1998 [125], and as an alternative means for the spread of JEV to Guam after the capture of *Cx. tritaeniorhynchus* mosquitoes in light traps set up on ships in the East China Sea [142]. The concept of biting insect dispersal by wind currents over long distances is not new and has been described and discussed in a number of instances, e.g., [106,143], and was recently the subject of modelling the dispersal of biting midges from the island of Timor as the possible means of introduction of bluetongue serotypes and genotypes into northern Australia [144].

The emergence and subsequent widespread incidence of JEV in eastern Australia strongly suggest that the GIV JEV strain will become established in year-round transmission cycles in northern Australia with occasional extension into southern regions when environmental conditions are suitable, as occurs with MVEV. Within Australia, waterbirds [67,133,145,146,147,148] and pteropid bats [92,93,94], who are highly mobile and often travel long distances to access resources, would probably be the most likely disseminators of viruses over the continent and also be participants in local transmission cycles together with feral and domestic pigs. Satellite-tracking studies have shown that fruit bats can fly up to 80 km for food, and travel up to 6000 km annually between a network of roosts, but with no uniformity among their directions of travel [94]. Knowledge of the geographic incidence of MVEV would suggest that JEV should also be present in the north of Western Australia, particularly in the Kimberley Region, where MVEV is endemic with annual virus isolations from mosquitoes, or will spread there in the near future; to date, no evidence of its presence in Western Australia has been detected in either mosquitoes or sentinel chickens, nor in feral pigs although it is probable that the limited feral pigs investigations were too late in the season for any continued virus activity. Whether JEV will persist in SE Australia is an open question, but there is no strong evidence that MVEV persists in SE Australia, and it is believed that JEV GIV viruses are tropical rather than more temperate in their occurrence. Nevertheless, it is an important question that needs to be addressed.

Successful establishment requires the presence of vertebrate hosts and competent vector species; Australia has both in abundance. There are sufficient numbers of potential vertebrate hosts in water birds, feral and domestic pigs and Pteropid bats to drive transmission. *Cx. annulirostris* is the major indigenous vector of MVEV and WNV in Australia, and is a proven vector of JEV in the Torres Strait and northern Queensland [30,31,34,98]. This species is found widely in Australia and can be highly abundant when conditions are suitable, which drives intense transmission of MVEV and WNV [149]. It has previously been postulated that different lineages of *Cx. annulirostris* may vary in their vector competence for different genotypes of JEV or that they feed on vertebrate hosts which do not produce high viraemia levels, such as macropods [150,151]. Both factors may help to explain why JEV did not become established during previous incursions [6]. However, the scale of JEV in 2021–2022 suggests that the most widespread mainland lineage of *Cx. annulirostris* could be a highly efficient vector of the G4 JEV. In addition, the explosion in wading bird and feral pig populations following multiple years of above average rainfall may have provided a large pool of blood meal hosts for *Cx. annulirostris* to feed upon.

Other JEV vectors including *Cx. gelidus*, *Cx. triteniorhynchus*, *Cx. sitiens* and *Cx. palpalis* are not as widespread, but may play a role in JEV transmission where they are locally abundant, as may members of the *Cx. pipiens* group, exemplified by *Cx. quinquefasciatus*, in specific areas such as piggery waste water. There is also a strong possibility that over-wintering of the virus could occur in northern Australia via vertical transmission in desiccation-resistant eggs of the *Aedes* species, as described for MVEV [152]. Clearly, there are many questions that need to be answered regarding the fitness of the G4 JEV in mosquitoes and the bionomics of incriminated vectors that affect their interaction with vertebrate hosts. Whether JEV will persist in SE Australia remains to be determined, although limited evidence would suggest that it may be unlikely—MVEV does not appear to persist so far south, and GIV has been a tropical genotype until this outbreak.

There are three other closely related members of the Japanese encephalitis sero-complex co-circulating in Australia: MVEV, WNV and Alfuy virus (ALFV) [153]. There is no evidence to indicate that these three flaviviruses interfere with each other’s replication in Australia, despite a similar ecology in which they are thought to share vertebrate hosts and vector species. It is not known whether they will prevent or interfere with JEV replication or its transmission cycles, such as by competition for non-immune vertebrate hosts and the production of cross-protective immunity in these hosts [154], but this needs to be determined. Elsewhere, co-circulation of closely related JE sero-complex flaviviruses that share ecologies are known to occur in parts of Asia where JEV and WNV co-circulate [155,156], in parts of Europe where WNV and Usutu viruses co-circulate [157,158], and in the Americas were WNV and St Louis encephalitis virus co-circulate [159,160].

Climatic factors including heavy rainfall and flooding in the northern and eastern parts of the continent, along with flooding in central Australia, play an important role in virus spread, particularly in the genesis of MVEV outbreaks in eastern and south-eastern Australia. They have been associated with the La Niña phase of the El Niño–Southern Oscillation climate weather pattern of above-average winter–spring rainfall in Australia, particularly across the east and north of the Continent [161]. Australia has experienced two consecutive years of higher-than-average rainfall, 2020–21 and 2021–22, and record-breaking flooding across the east of the country in both years has been associated with a La Niña weather pattern. This may have assisted in the spread of JEV over the past year through a combination of greatly increased mosquito breeding sites and conditions, and the attraction of waterbird movements into fresh feeding opportunities.

## 7. Development of JEV Surveillance Strategies

If JEV becomes endemic in Australia, there needs to be a strong One Health approach to surveillance and mitigation strategies with seamless collaboration between animal, human and environmental jurisdictions by combining relevant information from sentinel animals, reservoir hosts, mosquito populations and breeding, environmental and climatic conditions and human infections. Surveillance measures in northern, western and eastern mainland Australia have largely been aimed at detecting MVEV using either sentinel chickens or virus detection in field-caught mosquitoes, combined with predictive modelling of climatic conditions [161,162], but have they failed to provide early evidence of JEV activity. Whether this was due to the placement of the sentinel chickens, a lack of sensitivity or insufficient scale of mosquito trapping is not known. However, the large areas covered in surveillance and the sparsity of the human population make arboviral surveillance an expensive and difficult undertaking and requires careful planning for placement of sentinel chickens, carrying out regular mosquito trapping or other forms of sentinel animal surveillance to mitigate these problems of distance. It is also essential that the serological and diagnostic tests are fit for purpose—that is, that serological tests are sensitive and specific for JEV, regardless of genotype, and that molecular assays are sensitive and specific for JEV GIV viruses. The various diagnostic tests available have been reviewed in detail elsewhere, with their relative merits and their advantages and disadvantages [163].

The use of sentinel chickens in JEV surveillance elsewhere has had mixed results; in some studies, chickens have been relatively useful [164,165], whereas in others seroconversion rates were too low or absent [30,166,167,168]. Sentinel chicken surveillance linked to rainfall data have proven to be an effective early warning of MVEV activity and to inform on risk management [160]. Sentinel chicken flocks are used in Western Australia, NSW and Victoria for MVEV and WNV surveillance [169], but it is suggested that more sentinel flocks could be located at sites close to piggeries, near effluent ponds and, where feasible, near to major waterbird breeding sites to provide better opportunities of JEV detection.

Sentinel pigs have been used successfully in the surveillance of JEV in north Queensland and the Torres Strait, but their deployment has to be well away from human habitation because of their role in JEV amplification. However, they are expensive to maintain and to sample, and have been discontinued in Australia [43]. In contrast, pigs have been used successfully in JEV surveillance in many countries, e.g., [170,171,172,173], but are perhaps most effective in temperate climates as an early warning of an impending epidemic, or in tropical and sub-tropical areas where JEV is absent or rare, so further consideration of their value in Australia is warranted.

Sero-surveillance of piggeries played a major role in investigations to determine the extent of JEV transmission in the 2022 outbreak in south-eastern Australia, and to identify earlier evidence of JEV through retrospective testing. The high turn-over of grower pigs in piggeries (average slaughter age is 6 months) provides an ongoing source of potential amplifier host animals. Although a maternal antibody can persist for 3–4 months of age, weaned piglets become susceptible to JEV infection thereafter and detection of specific antibody responses to JEV can be considered a reliable indicator of natural exposure. While cross-reactive antibodies to MVEV and WNV can confound serological testing, identifying specific responses to JEV can provide an indication of prior JEV activity within a piggery. Sporadic serological testing of feral pigs and bovines in northern Australia has also been valuable for detecting the presence of flaviviruses, including JEV, as part of the NAQS program for many years. However, differentiating specific antibody responses is particularly challenging in areas where antigenically related flaviviruses, such as MVEV and WNV, are also active, and where animals may experience multiple exposures throughout their lifetime, which can result in broad, cross-reactive antibody responses. In combination with serology, destructive sampling of feral pigs for nucleic acid detection by PCR testing of blood and tissue samples has been very successful and specific for detecting JEV in northern Australia during the current outbreak [56].

Wherever domestic pigs are kept, regular sampling by non-invasive methods, such as oronasal swabs or oral fluids from rope chewing, should also be considered. JEV was found to persist in the tonsils of experimentally infected pigs for at least 25 days, long after the viraemic phase and despite the presence of high levels of neutralizing antibodies [173], and could be shed in oronasal secretions [174,175,176]. Recently it has been shown that PCR testing of oronasal secretion samples could be employed as a non-invasive, alternative method of implementing JEV surveillance in an epidemic area prior to the detection of virus-positive mosquitoes [176,177]. Such a combination of sampling and molecular-based assays may remove some of the ambiguities associated with cross-reactivity of flaviviruses in serological tests typically used as part of sentinel surveillance systems. Another possible surveillance method for use in domestic piggeries could be the detection of JEV in piggery effluents. JEV genomes or genomic fragments have been reported in the faeces of infected pigs [178], but it is uncertain whether the live virus is also present. JEV has also been reported in human urine from JE cases by deep sequencing [179] and by PCR and virus isolation in culture [180]. A number of other flaviviruses have been detected in urine from humans and various animals, including Zika, dengue and two additional members of the Japanese encephalitis serological complex, WNV and MVEV [181,182,183,184,185,186], which demonstrates that JEV in urine should not be an unexpected finding. While there has not yet been confirmation of the presence of JEV or JEV-specific genomic fragments in pig urine they would, if present in the effluent, provide an indication of active virus transmission in the piggery and provide a possible novel surveillance method.

A number of other sentinel animal systems have been used for JEV surveillance with varying degrees of success, including dogs [187,188] and goats [189,190,191]. Cattle have been used and are particularly attractive to mosquitoes; however, as long-lived hosts, once seropositive, they are of little further use as sentinels [187]. JEV seroprevalence in both cattle and goats were shown to be better predictors of human infection risk than porcine seroprevalence [189]. In all sentinel animal systems, once an animal seroconverts, there is no further use for that animal as a sentinel, and there are significant costs in supplying and maintaining a sentinel animal system, along with resourcing for associated laboratory testing and maintaining animal ethics requirements. Nevertheless, it is proposed that if a sentinel system is required, then goats could be considered as possible sentinel hosts in Australia as they are more sensitive for sero-surveillance than pigs and do not present a health risk, so they can be used close to human habitation.

Mosquito trapping for virus detection and/or identification is a highly effective surveillance method [192] and should be significantly boosted, particularly around piggeries and their effluent ponds, as well as in waterbird habitats. Mosquito-based surveillance for JEV in Australia was historically conducted by pooling trapped mosquitoes by species and testing them using virus isolation or molecular assays [31,34]. Whilst this does provide important information on virus carriage rates in different mosquito species, it is laborious and can take a considerable amount of time to process traps which can potentially contain 10,000s of mosquitoes. Thus, alternative systems have been developed to expediate mosquito-based surveillance, which can include processing mosquitoes collected over a 2-week period in large pool sizes [193,194], detection of viral RNA in saliva expectorates by mosquitoes [195,196] or in mosquito excreta [197,198]. Although the latter two methods have not detected JEV in field studies, they have detected MVEV and WNV in field populations of mosquitoes, and thus should be suitable for detection of JEV.

To determine temporal transmission dynamics and identify periods of risk, mosquito-based JEV surveillance should be conducted over the entire season in which mosquitoes are active. Once trapping has been conducted across several years, it may be possible to identify locations where virus activity is regularly detected and that could serve as indicator sites for impending transmission. Irrespective of the mode/s of surveillance, its goal should be to recognize JEV activity before human cases occur so that appropriate public health and animal health measures can be implemented.

## 8. Control of JEV in the Australian Context

A number of strategies exist for the control of JEV transmission and prevention of human cases, ranging from vaccination to vector control and to personal protective measures. JEV has been shown to exist as a single serotype [22], although antigenic heterogeneity has been reported between different isolates and genotypes, e.g., [24]. There are no effective antivirals for JEV [50,199,200], but several promising anti-JEV candidate compounds have been identified in animal models or in vitro experimentation [50], and some promising candidates are under investigation in clinical trials [201]. Two vaccines against JEV are licensed for use in Australia: IMOJEV, a live attenuated chimeric vaccine comprising the yellow fever virus 17D vaccine strain as the backbone and the pre-membrane and envelope genes of the GIII Chinese live attenuated JEV vaccine, SA-14-14-2 strain [202]; and JESPECT (IXIARO in North America and Europe), an adjuvanted inactivated Vero cell-grown vaccine using the SA-14-14-2 strain [203]. The SA-14-14-2 strain is an attenuated GIII strain [204] and has been shown to provide protection against the different genotypes of JEV, e.g., [23,205,206], with the possible exception of genotype V viruses, which are significantly different at both nucleotide and amino acid levels [14,207,208,209,210]. While vaccination was shown to be effective in JEV protection in the Torres Strait since its implementation in late 1995 [211], it is difficult to determine who should be at the forefront for receiving the vaccine in Australia under current circumstances. It would be expected that those most at risk might be: piggery workers; abattoir workers; hunters of feral pigs; people living nearby to piggeries or wetlands; laboratory workers; park rangers including First Nations sea and land rangers in northern Australia; and campers, fishers and others involved in outdoor activities in areas where infected mosquitoes might be expected to occur, such as near lakes, lagoons and wetlands. It is not known whether prior exposure to MVEV would provide some protection against JEV infection. A single dose of IMOJEV, however, gives complete protection against MVEV challenge in mice [212].

At the time of writing, there are no registered vaccines for use in animals in Australia. In other countries, vaccination of pigs and horses has been used successfully to control disease in these animals. In JEV endemic areas, vaccination of pigs may also be effective in reducing the risk of human disease [213]. However, vaccination of pork production animals has not been applied widely due to high costs associated with immunizing large numbers of newborn pigs, their high turn-over and the short period for effective immunization, which may be limited by the presence of maternal antibodies [214]. Nevertheless, annual vaccination of sows prior to peak seasonal JEV activity is used in some countries such as South Korea and Taiwan to prevent or limit production losses from reproductive disease caused by JEV infection [215,216]. Annual vaccination of high value horses, such as racehorses or thoroughbreds, is also undertaken in several countries where JEV is found (e.g., Japan, China, Malaysia and Singapore) or in racehorses that travel from JE-free locations to JEV-endemic countries to compete in international racing events [77,217]. Vaccination of horses has been a very effective means at reducing levels of equine JE in Japan, South Korea and Hong Kong [26,77,218].

There are several GIII-based veterinary vaccines available, based on the Nakayama, Beijing, BMIII, AT222, M or Anyang strains, and produced as either inactivated or live vaccines [77]. The emergence of other genotypes in Asia has led to a number of studies that have evaluated the efficacy of GIII-based vaccines against heterotypic circulating strains. Much of this work has focused on the displacement of GIII by GIb viruses [18]. Protective levels of neutralizing antibody responses to heterotypic JEV strains have been reported from sera collected from horses and pigs immunized with inactivated and live-attenuated GIII vaccines, e.g., [28,206,215]. However, significant differences in the levels of cross-neutralizing antibodies to heterotypic JEV strains were consistently found. Only limited in vivo studies have been performed to address vaccine efficacy against heterotypic JEV infection. A recent study conducted on pig farms in Taiwan found that a GIII AT222 live-attenuated vaccine was less effective at preventing stillbirth and abortion in naïve gilts (females that have not given birth to a litter of piglets) caused by circulating GI compared to GIII virus [29]. Further research is urgently needed to evaluate the efficacy of GIII-based veterinary vaccines to prevent JE disease caused by the Australian GIV strain.

Control measures targeting the mosquito vectors also have the potential to limit JEV transmission [219]. Unfortunately, the widespread geographical distribution of *Cx. annulirostris* larval habitats impacts the feasibility of broadscale application of larvicides for the control of this species, as is the case with other floodwater *Culex* around the world. Similarly, the logistics associated with space-spraying adulticides across such a vast area and the limited efficacy for sustained population reduction compromise this as a control strategy. However, some focal mosquito population reduction via larval and adult control could be achieved proximal to pig farms, and guidelines on best practice for these activities have been formulated [220]. A number of personal protective measures are promoted to limit contact with mosquitoes and include avoiding risk areas at and between dawn and dusk (when *Cx. annulirostris* is most active), wearing loose-fitting long-sleeved clothing, applying repellent formulations containing the active ingredients DEET (N,N-diethyl-3-methylbenzamide), picaridin or oil of lemon eucalyptus (p-menthane-3,8-diol), and ensuring insect screens in houses, caravans and tents are functional to prevent mosquito entry [221].

## 9. Conclusions

There is little doubt that suitable vertebrate hosts and mosquito vectors exist in Australia to ensure that ongoing transmission cycles of JEV GIV can be maintained in the Australian ecosystem, which increases the potential for the virus to become endemic in tropical northern Australia, if it has not already done so. It is highly likely that JEV will have a similar ecological pattern in mainland Australia to those of the indigenous members of the Japanese encephalitis serological complex, MVEV, WNV, and ALF virus, with respect to geography, vectors and avian hosts. There remains much to determine about the probable JEV transmission cycles, such as the identity of the major avian vertebrate hosts, and the potential roles of feral pigs and fruit bats species. In addition, although some Australian mosquito species are known to be competent vectors, there are a number of others that might be important in specific ecological situations.

The origin and route by which the JEV GIV virus reached Australia and where it entered northern Australia remains to be determined, and it may be difficult to obtain an absolute answer. Most evidence would suggest that the virus probably came from a focus of activity in the Indonesian archipelago or PNG through either wind-blown mosquitoes, or through vagrant or migratory birds. The PNG-Torres Strait-Cape York flyway is an obvious possibility, especially given the abundance of feral pigs in north-eastern Queensland, and the occurrence of sero-positive pigs in the Torres Strait and more recently in the NPA. Further information on the incidence of JEV GIV in Western Province, PNG, is urgently needed and might give credence to the Torres Strait flyway. Nevertheless, other possible origins, such as from Timor Leste, cannot be discounted. Wind-blown birds caught up in monsoonal weather patterns and JEV infected mosquitoes entering through harbouring on planes or boats are also possibilities. The length of viraemia in birds is believed to be between 3 and 6 days, so this might place some barriers on distance.

The emergence of JEV GIV in Australia, the rarest of the JEV genotypes, was the third of the five known genotypes to occur in Australia, and as such demonstrates the ongoing risk of further incursions in the future, as well as the need to maintain an ‘over the horizon’ surveillance program.

The antigenic similarities and the involvement of flaviviruses in original antigenic sin necessitates a reassessment of the current serological techniques employed in Australia, both with respect to case diagnostics and in surveillance procedures. It may also be necessary to reassess surveillance procedures to account for some of the known ecological differences with MVEV and WNV, such as swine involvement with JEV.

Little is known of the properties of JEV GIV. All previous, confirmed isolates had only been reported from Indonesia, and only in the years 1980–81 and 2017–19, so it is assumed that the virological and pathogenic properties will be the same or similar to those determined for GI and GIII viruses in other parts of Asia, but this may be erroneous given that differences have been reported between GI and GIII viruses. Interestingly, the most divergent GV viruses appear to be more virulent than other GI and GIII viruses in mice, and also appear to have different vector preferences than other genotypes [210]. The 2021 Tiwi Island case and the earlier Bali case in which the patient died in Australia, would suggest that the pathology of human cases may not vary greatly between these two cases and those described for the other genotypes, but we have limited data to make this assessment. We also do not yet know the frequency of severe disease resulting from JEV GIV infection. While the widespread infection and reproductive disease observed in pigs during the 2022 outbreak in south-eastern Australia may reflect an immunologically naïve and susceptible population, the outbreak strain may encode determinants of enhanced virulence or pathogenesis compared to earlier Australia JEV isolates. It is also unclear whether the Australian GIV strain has displaced the previously detected Australian genotypes (I and II). Such a scenario raises questions about the biological mechanisms underlying the emergence of a dominant strain in the region.

This present outbreak has shown that we have yet to develop governance structures that allow the timely exchange and integration of the detailed data needed to maximise the benefits of a One Health approach. Early steps have been taken to address this, and it is hoped that these will lead to a robust and enduring cross-sectoral collaboration. The next year will potentially provide an indication of JEV endemicity in northern Australia. As occurred in the past two years, the Southern Oscillation Index forecast is for a La Niña weather pattern that is predicted to result in heavy, widespread rainfall in the northern and eastern areas of the continent and, given the effects of the two previous La Niña years, will probably lead to extensive flooding. Further time will be required to better understand the virus ecology, including the principal vectors and vertebrate hosts present in different ecosystems in Australia.

## Figures and Tables

**Table 1:** All known isolates of Japanese encephalitis virus genotype 4 from 1979–2021.

Virus	Origin	Year	Source	GenBank Access. No.	Refs.
VN113	Vietnam	1979	Human	KU705228.1	Unpublished
JKT8442	Bali	1980	*Culex tritaeniorhynchus*	L42159	[12,44]
JKT6468	Flores	1981	*Cx. tritaeniorhynchus*	AY184212	[12,44]
JKT7003	Java	1981	Unidentified mosquito species ^a^	L42161	[12,44]
JKT7089	Bantul, Java	1981	*Cx. vishnui*	JQ429309	[12,44]
JKT7180	Central Java	1981	*Cx. tritaeniorhynchus*	JQ429310	[12,44]
JKT7887	Java	1981	Unidentified mosquito species	L42160	[12,44]
JKT9092	Bali	1981	Unidentified mosquito species	L42158	[12,44]
JEV/sw/Bali/93	Bali	2017	Pig serum	LC461960	[45]
JEV/sw/Bali/94	Bali	2017	Pig serum	LC461961 ^b^	[45]
19CxBa-83-Cv	Bali	2019	*Cx. vishnui*	LC579814	[46]
JEVBali2019	Bali/Australia	2019	Human	MT253731.1	[47]
JE(Tiwi)2021 ^c^	Tiwi Is, Australia	2021	Human	OM867669	[36,48]

^a^ The species composition of the virus positive pool was not reported. ^b^ Genome sequence incomplete. ^c^ Full sequence obtained, but no infectious virus isolated.

## Data Availability

Not applicable.
